# Factors for poor oral health in long-term childhood cancer survivors

**DOI:** 10.1186/s12903-023-02762-0

**Published:** 2023-02-04

**Authors:** Tushar Patni, Chun-Teh Lee, Yimei Li, Sue Kaste, Liang Zhu, Ryan Sun, Melissa M. Hudson, Kirsten K. Ness, Ana Neumann, Leslie L. Robison

**Affiliations:** 1 Department of Biostatistics, St. Jude Children’s Research Hospital, Memphis, TN USA; 2 Department of Periodontics and Dental Hygiene, The University of Texas Health Science Center at Houston School of Dentistry, Houston, TX USA; 3 Department of Internal Medicine, The University of Texas Health Science Center at Houston, Houston, TX USA; 4 Department of Biostatistics, MD Anderson Cancer Center, Houston, TX USA; 5 Department of Epidemiology, St. Jude Children’s Research Hospital, Memphis, TN USA; 6 Department of Oncology, St. Jude Children’s Research Hospital, Memphis, TN USA; 7 Department of General Practice and Dental Public Health, The University of Texas Health Science Center at Houston School of Dentistry, Houston, TX USA; 8 Department of Diagnostic Imaging, St. Jude Children’s Research Hospital, Memphis, TN USA

**Keywords:** Cohort studies, Dental care, Dental caries, Mouth diseases, Periodontal diseases, Radiation effects

## Abstract

**Background:**

Survivors of childhood cancer are at risk for therapy-related dental diseases. The purpose of the study was to investigate the associations between clinical, socioeconomic, and demographic factors and oral diseases in the St. Jude Lifetime Cohort (SJLIFE) participants.

**Methods:**

We performed a retrospective medical chart review and evaluated longitudinal self-reported dental outcomes in 4856 childhood cancer survivors and 591 community controls participating in the St. Jude Lifetime Cohort (SJLIFE) study. Univariate and multivariable logistic regression models were used to assess the impact of socioeconomic factors, treatment exposures and patient demographics on dental outcomes.

**Results:**

Cancer survivors were more likely to report microdontia (odds ratio (OR) = 7.89, 95% confidence interval (CI) [4.64, 14.90]), abnormal root development (OR = 6.19, CI [3.38, 13.00]), hypodontia (OR = 2.75, CI [1.83, 4.33]), enamel hypoplasia (OR = 4.24, CI [2.9, 6.49]), xerostomia (OR = 7.72, CI [3.27, 25.10]), severe gingivitis (OR = 2.04, CI [1.43, 3.03]), and ≥ 6 missing teeth (OR = 3.73, CI [2.46, 6.00]) compared to controls without cancer history. Survivors who received classic alkylating agents (OR = 1.6, CI [1.36, 1.88]), anthracycline antibiotics (OR = 1.22, CI [1.04, 1.42] or radiation therapy potentially exposing the oral cavity (OR = 1.48, CI [1.26, 1.72]) were more likely to report at least one dental health problem after controlling for socioeconomic factors, age at last follow-up and diagnosis, other treatment exposures, and access to dental services. Survivors who had radiation therapy potentially exposing the oral cavity (OR = 1.52, CI [1.25, 1.84]) were also more likely to report at least one soft tissue abnormality after controlling for socioeconomic factors, age at last follow-up and diagnosis, other treatment exposures, and access and utilization of dental services.

**Conclusions:**

Childhood cancer survivors have a higher prevalence of oral-dental abnormalities than the controls without a cancer history. Cancer treatment, socioeconomic factors, and access to oral health care contribute to the prevalence of dental abnormalities.

## Background

Childhood cancer survivors experience adverse medical and psychosocial late effects resulting from cancer treatments during vulnerable periods of physical development. Dental late effects can occur following cancer treatment during early childhood, a period associated with tooth development or odontogenesis [1]. Chemotherapy’s interferences in the odontogenesis process results from disruption with cell cycle events and intracellular metabolism can alter patterns of ameloblastic reproduction, secretory function, membrane permeability, calcium exchange across the cell membrane, or odontoblastic activity [2, 3]. Radiotherapy potentially exposing dental structures can alter oral integrity by damaging the tooth bud or underlying soft tissues, or by causing salivary gland dysfunction, resulting in xerostomia and hyposalivation [4–7]. Salivary gland injury and abnormal craniofacial and dental development can directly or indirectly cause oral diseases. Several studies have reported a higher prevalence of caries, gingivitis, and xerostomia in childhood cancer survivors compared to individuals without a history of cancer [8]. However, large cohort studies comprehensively investigating associations between oral disorders and potential risk factors in childhood cancer survivors are limited.

This study aimed to assess associations between clinical, socioeconomic and demographic factors and oral diseases in the St. Jude Lifetime Cohort (SJLIFE) participants, a large childhood cancer survivor cohort. The medical and dental data collected by SJLIFE provide an opportunity to characterize the oral disease burden in long-term survivors and identify risk factors associated with cancer treatment related sequelae. Data from this study can inform potential interventions to prevent oral health sequelae resulting from childhood cancer treatment in the early stage [9].

## Methods

### Study population

Participants included members of the SJLIFE, a retrospective cohort study with prospective follow-up and ongoing accrual of patients diagnosed and treated at St. Jude Children’s Research Hospital (SJCRH) over five decades (1962–2012). The study was initiated in 2007 to characterize long-term health outcomes among individuals who survived at least five years post diagnosis for childhood cancer at SJCRH and who had been diagnosed with cancer through 30th June 2012. The acquisition of data happened for events (dental health outcomes) that occurred prior to and after study enrollment in SJLIFE. Informed consent was obtained for participation in the study [10]. Age-, sex-, race-matched control participants without a history of childhood cancer were recruited from non-first-degree relatives of survivors or the community and completed the same clinical assessments as the survivor [10]. SJLIFE participants are invited to return to SJCRH at least once every 5 years for protocol-based medical evaluations and assessments of neurocognitive function, physical performance status and patient-reported outcomes [10]. All subjects who fulfilled these criteria and were willing to participate were included in this study.

Survivors’ medical records were abstracted to obtain detailed treatment exposures including cumulative doses of chemotherapeutic agents, radiation fields and doses, surgical procedures, and hematopoietic cell transplantation. The following treatment variables were included in our analysis: total body irradiation, radiation potentially exposing the oral cavity, and cumulative doses of alkylating agents (bendamustine, busulfan, carmustine, chlorambucil, cyclophosphamide, ifosfamide, lomustine, mechlorethamine, melphalan, procarbazine, and thiotepa), and anthracycline antibiotics (daunorubicin, liposomal daunorubicin, doxorubicin, liposomal doxorubicin, epirubicin, idarubicin, and mitoxantrone). We chose these variables because anthracycline antibiotics are strongly associated with the prevalence of dental defects [11], and the remaining variables were chosen based on the report from Childhood Cancer Survivor Study [5] which also investigated the association of these variables with dental abnormalities. Dental health outcomes self-reported by participants using a questionnaire included hypodontia, microdontia, enamel hypoplasia, abnormal root development, ≥ 6 missing teeth, denture use, root canal, dental bridge use, palatal lift prosthesis use, xerostomia, gingivitis, and ≥ 5 cavities (Table 1). Questionnaire items are drawn from published and validated scales or index items from previous surveys such as the Childhood Cancer Survivor Study^5^ or Behavioral Risk Factor Surveillance System [12]. The subjects were provided with a helpline number in the survey to get any assistance for completing the questionaries. Since these conditions were recorded at multiple time points, we transformed the data to cross-sectional data by observing that whether the subject had ever experienced these conditions or not during the follow-up period. We followed the same procedure for age, socioeconomic and other factors except for demographics factors which were recorded at baseline. Combination measures evaluated included the presence of ≥ 1 of the following dental outcomes as “≥ 1 dental health problem”: ≥ 1 teeth with hypodontia, microdontia, enamel hypoplasia, or abnormal root development and/or ≥ 6 missing teeth, and the presence of ≥ 1 of the following as “soft tissue problem”: xerostomia and/or severe gingivitis. Socioeconomic and demographic factors considered to control for any confounding effect in the analysis were sex, race, educational attainment, previous year household income, ever had dental insurance, ever had health insurance, ever smoked, ever had teeth cleanings, ever had difficulty finding dentists, and ever had dental visits.

**Table 1:** Dental health and care questions in the SJLIFE survey

Questions	Abbreviation
Had one or more missing teeth because they did not develop?	mistth
Had a lack of or decreased amount of enamel on surface of teeth (hypoplasia)?	enameldef
Had abnormal shaped (small or malformed) teeth?	abntth
Had abnormal root development?	abnrt
Had difficulty in producing saliva (dry mouth) that required treatment such as artificial saliva?	drymth
Had severe gingivitis or gum disease requiring surgery or deep cleaning?	gumdis
Had root canal therapy?	rtcanl
Had more than 5 cavities?	cavities
Lost 6 or more teeth due to decay or gum disease?	lost6th
Worn a dental bridge (for missing or removed teeth)?	dntbrg
Worn removable dentures (complete or partial upper or lower or both)?	dentur
Worn a prosthesis to lift your palate to improve the quality of your voice?	dntpros

### Statistical analysis

Descriptive statistics of the demographic, socioeconomic, cancer diagnoses, and treatment characteristics were calculated. For each variable, the frequency of missing values was less than 10% and as a result, we didn’t perform any data imputation and sensitivity analysis. We used Chi-squared/Wilcoxon rank sum tests to investigate whether these factors differed between survivors and controls. We adjusted *P* values according to false discovery rate corrections and obtained *q* values when appropriate.

We compared the prevalence of dental outcomes between survivors and controls, associations between demographic, socioeconomic and dental service factors and dental outcomes, and associations between demographic and socioeconomic factors and receipt of dental services in the entire cohort using logistic regression. If the association between a specific dental outcome differed between survivors and controls (*P* < 0.05), we evaluated demographic, socioeconomic, dental service, and treatment related risk factors for that outcome in univariate and multivariable models, retaining variables for the multivariable regression where p-values were < 0.10 in univariate models. All analyses were conducted with R software (version 3.6.2).

## Results

**Fig. 1:**
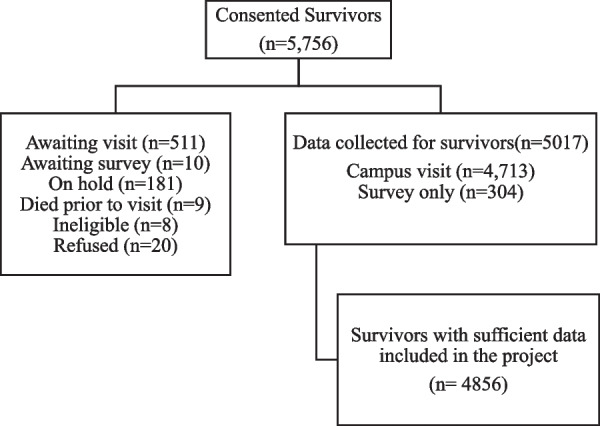
Flowchart of the number of survivors consented for the study

5756 survivors and 625 community controls consented to participate in the study, but only 4856 survivors and 591 community controls were included due to following reasons: died prior to visit, did not participate in the survey, ineligible etc. The flowchart in Fig. 1 shows the breakdown of various reasons due to which survivors were not included in this study. Data collected for 5017 survivors (campus visit + survey only) but only 4856 of them were used in the final analysis because 161 subjects didn’t have information for most of the socio-economic and treatment variables which were investigated in this study. The survivor group had a higher percentage of male, nonwhite, and younger persons than the control group (Table 2). Controls were more likely to achieve more advanced education compared to survivors.

**Table 2:** Characteristics of survivors and community controls

Characteristic	Community control, N = 591^a^	Survivor, N = 4856^a^	*P* value^b^	q value^c^
Age at last follow-up	32 (25, 41)	31 (23, 39)	< 0.001	< 0.001
Gender			< 0.001	< 0.001
Female	334 (57%)	2345 (48%)		
Male	257 (43%)	2511 (52%)		
Race			< 0.001	< 0.001
Non-white	71 (12%)	867 (18%)		
White	520 (88%)	3989 (82%)		
Household income			< 0.001	< 0.001
< $20, 000 per year	51 (9.4%)	738 (17%)		
> $20, 000 per year	492 (91%)	3637 (83%)		
Unknown	48	481		
Education			< 0.001	< 0.001
No post high school	86 (15%)	1599 (36%)		
Post high school	474 (85%)	2886 (64%)		
Unknown	31	371		
Dental insurance			0.2	0.3
Yes	411 (72%)	3241 (70%)		
No	157 (28%)	1402 (30%)		
Unknown	23	213		
Health insurance			0.6	0.7
Yes	512 (88%)	4239 (88%)		
No	71 (12%)	553 (12%)		
Unknown	8	64		
Smoking status			0.8	0.8
Yes	181 (31%)	1423 (30%)		
No	407 (69%)	3291 (70%)		
Unknown	3	142		
Dental visit			0.3	0.3
Yes	401 (68%)	3357 (71%)		
No	186 (32%)	1398 (29%)		
Unknown	4	101		
Dental cleaning			0.15	0.2
Yes	395 (67%)	3057 (64%)		
No	191 (33%)	1691 (36%)		
Unknown	5	108		
Difficulty finding dentist			< 0.001	< 0.001
Yes	1 (0.2%)	310 (6.7%)		
No	570 (100%)	4334 (93%)		
Unknown	20	212		

^a^Median (IQR); n (%)

^b^Wilcoxon rank sum test; Pearson's Chi-squared test

^c^False discovery rate correction for multiple testing

The median survivor age at cancer diagnosis was 6.7 (range 0–24.8) years and the median age at the time of the study was 31 (range 7.3–69.8 years) years (Table 3). The median time period between diagnosis and the last follow-up was 22 (range 5.9–55.5) years. The subjects were given chemotherapy/radiation within 5 years of primary cancer diagnosis. The median cumulative dose of cyclophosphamide is 6868.08 (range 300–38,868.41) mg/m^2^ and doxorubicin is 180 (range 15.78–723.95) mg/m^2^.

**Table 3:** Characteristics of cancer diagnosis treatment

Characteristic	N = 4856^a^
Age at diagnosis	
(0,5]	1991 (41%)
(5,10]	1084 (22%)
(10,15]	1017 (21%)
(15,20]	722 (15%)
(20,25]	42 (1%)
Diagnosis group	
Bone cancer	293 (6.0%)
Central nervous system (CNS)	659 (14%)
Germ cell tumor	100 (2.1%)
Histiocytosis	37 (0.8%)
Hodgkin lymphoma	524 (11%)
Leukemia	1671 (34%)
Liver malignancies	32 (0.7%)
Melanoma	35 (0.7%)
Nasopharyngeal carcinoma	31 (0.6%)
Neuroblastoma	225 (4.6%)
Non-Hodgkin lymphoma	314 (6.5%)
Non-malignancy	14 (0.3%)
Others	61 (1.3%)
Retinoblastoma	255 (5.3%)
Soft tissue sarcoma	310 (6.4%)
Wilms tumor	295 (6.1%)
Total body irradiation	
Yes	136 (2.8%)
No	4656 (97%)
Unknown	64
Radiation with potential impact to oral cavity	
Yes	1957 (40%)
No	2899 (60%)
Patients ever received anthracyclines	
Yes	2787 (57%)
No	2069 (43%)
Patients ever received classic alkylating agents	
Yes	2873 (59%)
No	1983 (41%)
Year of diagnosis	
(1958,1965]	18 (0.4%)
(1965,1972]	157 (3.2%)
(1972,1979]	478 (9.8%)
(1979,1986]	799 (16%)
(1986,1992]	910 (19%)
(1992,1999]	1006 (21%)
(1999,2006]	978 (20%)
(2006,2013]	510 (11%)

^a^Median (IQR); n (%)

### Comparison to control cohort

Survivors were more likely to report dental problems than were controls (Table 4), including misshapen teeth (abnth) (14% survivors, 2.1% controls), abnormal tooth roots (abnrt) (8.9% survivors, 1.6% controls), missing teeth (mistth) (10% survivors, 4% controls), enamel deficits (enameldef) (17% survivors, 4.6% controls), xerostomia (drymth) (5% survivors, 0.7% controls), severe gingivitis/periodontitis (gumdis) (10% survivors, 5.3% controls), and > 6 missing teeth (lost6th) (12% survivors, 3.6% controls). Survivors also reported a high frequency of > 5 carries (cavities), root canal therapy (rtcanl), dental bridges (dntbrg), removable dentures (denture), and palatal lift prosthesis (dntpros), but these percentages did not significantly differ between survivors and controls.

**Table 4:** Comparison of dental outcomes between survivors and community control

Characteristic	No^a^	Yes^a^	N	OR	95% CI	*p* value	q value^b^
abntth			5177				
Community control	568 (98%)	12 (2.1%)		–	–		
Survivor	3940 (86%)	657 (14%)		7.89	4.64, 14.9	< 0.001	< 0.001
abnrt			5046				
Community control	568 (98%)	9 (1.6%)		–	–		
Survivor	4070 (91%)	399 (8.9%)		6.19	3.38, 13.0	< 0.001	< 0.001
mistth			5247				
Community control	559 (96%)	23 (4.0%)		–	–		
Survivor	4191 (90%)	474 (10%)		2.75	1.83, 4.33	< 0.001	< 0.001
enameldef			4909				
Community control	535 (95%)	26 (4.6%)		–	–		
Survivor	3605 (83%)	743 (17%)		4.24	2.90, 6.49	< 0.001	< 0.001
drymth			5294				
Community control	585 (99%)	4 (0.7%)		–	–		
Survivor	4469 (95%)	236 (5.0%)		7.72	3.27, 25.1	< 0.001	< 0.001
gumdis			5237				
Community control	550 (95%)	31 (5.3%)		–	–		
Survivor	4175 (90%)	481 (10%)		2.04	1.43, 3.03	< 0.001	< 0.001
rtcanl			5212				
Community control	460 (80%)	112 (20%)		–	–		
Survivor	3594 (77%)	1046 (23%)		1.20	0.97, 1.49	0.11	0.13
cavities			4983				
Community control	284 (53%)	247 (47%)		–	–		
Survivor	2,267 (51%)	2185 (49%)		1.11	0.93, 1.33	0.3	0.3
lost6th			5263				
Community control	558 (96%)	21 (3.6%)		–	–		
Survivor	4107 (88%)	577 (12%)		3.73	2.46, 6.00	< 0.001	< 0.001
dntbrg			5310				
Community control	554 (95%)	29 (5.0%)		–	–		
Survivor	4409 (93%)	318 (6.7%)		1.38	0.95, 2.08	0.11	0.13
dentur			5318				
Community control	561 (96%)	25 (4.3%)		–	–		
Survivor	4443 (94%)	289 (6.1%)		1.46	0.98, 2.27	0.076	0.11
dntpros			5299				
Community control	581 (100%)	1 (0.2%)		–	–		
Survivor	4693 (99%)	24 (0.5%)		2.97	0.63, 53.2	0.3	0.3

OR, odds ratio; CI, confidence interval

^a^n (%)

^b^False discovery rate correction for multiple testing

### Use of dental services

Among all participants, those with higher educational attainment and income were more likely to visit a dentist (Table 5, *P* < 0.001), more likely to receive dental cleanings (*P* < 0.001), and less likely to experience difficulty finding a dentist (*P* < 0.001) than were those with less education/income. Males were less likely to visit a dentist (*P* < 0.001), less likely to receive dental cleanings (*P* < 0.001), and more likely to experience difficulty finding a dentist (*P* > 0.05) than were female subjects. White participants were more likely to visit a dentist (*P* < 0.001), more likely to receive dental cleanings (*P* < 0.001), and less likely to experience difficulty in finding dentist (*P* < 0.001) than were nonwhite participants.

**Table 5:** Use of dental services

Characteristic	No^a^	Yes^a^	N	OR	95% CI	*p* value	q value^b^
	*Dental visit within last year*						
Household income			4826				
< $20, 000 per year	363 (47%)	405 (53%)		–	–		
> $20, 000 per year	1011 (25%)	3047 (75%)		2.70	2.31, 3.17	< 0.001	< 0.001
Unknown	210	306					
Education			4964				
No post high school	632 (39%)	988 (61%)		–	–		
Post high school	844 (25%)	2500 (75%)		1.89	1.67, 2.15	< 0.001	< 0.001
Unknown	108	270					
Gender			5342				
Female	677 (26%)	1946 (74%)		–	–		
Male	907 (33%)	1812 (67%)		0.70	0.62, 0.78	< 0.001	< 0.001
Race			5342				
Non-white	325 (36%)	583 (64%)		–	–		
White	1259 (28%)	3175 (72%)		1.41	1.21, 1.63	< 0.001	< 0.001
	*Difficulty finding a dentist*						
Household income			4726				
< $20, 000 per year	632 (86%)	100 (14%)		–	–		
> $20, 000 per year	3816 (96%)	178 (4.5%)		0.29	0.23, 0.38	< 0.001	< 0.001
Unknown	456	33					
Education			4851				
No post high school	1417 (91%)	145 (9.3%)		–	–		
Post high school	3148 (96%)	141 (4.3%)		0.44	0.34, 0.56	< 0.001	< 0.001
Unknown	339	25					
Gender			5215				
Female	2424 (94%)	150 (5.8%)		–	–		
Male	2480 (94%)	161 (6.1%)		1.05	0.83, 1.32	0.7	0.7
Race			5215				
Non-white	794 (90%)	88 (10.0%)		–	–		
White	4110 (95%)	223 (5.1%)		0.49	0.38, 0.64	< 0.001	< 0.001
	*Teeth cleaned within last year*						
Household income			4822				
< $20, 000 per year	454 (59%)	314 (41%)		–	–		
> $20, 000 per year	1185 (29%)	2869 (71%)		3.50	2.99, 4.11	< 0.001	< 0.001
Unknown	243	269					
Education			4959				
No post high school	774 (48%)	848 (52%)		–	–		
Post high school	981 (29%)	2356 (71%)		2.19	1.94, 2.48	< 0.001	< 0.001
Unknown	127	248					
Gender			5334				
Female	816 (31%)	1806 (69%)		–	–		
Male	1066 (39%)	1646 (61%)		0.70	0.62, 0.78	< 0.001	< 0.001
Race			5334				
Non-white	389 (43%)	516 (57%)		–	–		
White	1493 (34%)	2936 (66%)		1.48	1.28, 1.71	< 0.001	< 0.001

OR, odds ratio; CI, confidence interval

^a^n (%)

^b^False discovery rate correction for multiple testing

### Risk factors for poor oral health in survivors and controls

The results of our multivariable logistic regression (Table 6) suggest that increased risk of ≥ 1 dental health problem was higher among survivors when compared with community controls (OR 3.44, 95% CI [2.63, 4.57]), and among older participants (age at last follow-up) (OR 1.02, 95% CI [1.01, 1.03]), females (OR 1.26, 95% CI [1.01, 1.45]), those who reported their race as white (OR 1.33, 95% CI [1.09, 1.63]), those who ever smoked (OR 1.66, 95% CI [1.42, 1.93]), those not graduating from high school (OR 1.5, 95% CI [1.28, 1.75]), and among participants who reported having difficulty finding a dentist (OR 4.8, 95% CI [3.55, 6.58]). Survivors status (OR 3.04, 95% CI  [2.04, 4.74]), older age at follow-up (OR 1.06, 95% CI [1.05, 1.06]), female sex (OR 1.34, 95% CI [1.12, 1.62]), those who ever smoked (OR 1.31, 95% CI [1.07, 1.59]), having difficulty finding a dentist (OR 4.64, 95% CI [3.44, 6.24]), and dental visit in the last year (OR 1.37, 95% CI [1.09, 1.73]) were associated with having one or more soft tissue oral problem.

**Table 6:** Results of multivariable analysis of combined outcomes for survivors and community controls

Characteristic	At least one dental health issue			At least one soft tissue issue		
	OR	95% CI	*P* value	OR	95% CI	*P* value
Study population						
Community control	–	–		–	–	
Survivor	3.44	2.63, 4.57	< 0.001	3.04	2.04, 4.74	< 0.001
Race						
Non-white	–	–				
White	1.33	1.09, 1.63	0.005			
Household income						
< $20, 000 per year	–	–		–	–	
> $20, 000 per year	0.82	0.67, 1.01	0.056	0.97	0.74, 1.28	0.8
Gender						
Male	–	–		–	–	
Female	1.26	1.10, 1.45	< 0.001	1.34	1.12, 1.62	0.002
Education						
Post high school	–	–		–	–	
No post high school	1.50	1.28, 1.75	< 0.001	0.95	0.76, 1.18	0.6
Dental insurance						
Yes	–	–		–	–	
No	0.90	0.76, 1.06	0.2	1.14	0.91, 1.42	0.2
Dental cleaning						
Yes	–	–				
No	1.08	0.92, 1.27	0.4			
Smoking status						
No	–	–		–	–	
Yes	1.66	1.42, 1.93	< 0.001	1.31	1.07, 1.59	0.007
Difficulty finding dentist						
No	–	–		–	–	
Yes	4.80	3.55, 6.58	< 0.001	4.64	3.44, 6.24	< 0.001
Age at last follow-up	1.02	1.01, 1.03	< 0.001	1.06	1.05, 1.06	< 0.001
Dental visit						
No				–	–	
Yes				1.37	1.09, 1.73	0.008

OR, odds ratio; CI, confidence interval

### Risk factors for poor oral health in survivors

Among survivors only, multivariable analysis (Table 7) identified increased risk for ≥ 1 dental health problem who were female (OR 1.29, 95% CI [1.12, 1.54]) reported their race as white (OR 1.25, 95% CI [1.02, 1.55]), had ever smoked (1.79, 95% CI [1.53, 2.1]) did not graduate from high school (OR 1.43, 95% CI: [1.18, 1.63])¸ were older at follow-up (OR 1.02, 95% CI [1.01, 1.03]), and had difficulty finding a dentist (OR 4.53, 95% CI [3.32, 6.25]). Survivors who were diagnosed at an older age had decreased risk for ≥ 1 dental health problem (OR 0.93, 95% CI [0.91, 0.94]).Survivors who received radiation therapy potentially exposing the oral cavity (OR 1.48, 95% CI [1.26, 1.72]) and who were treated with classic alkylating agents (OR 1.6, 95% CI [1.36, 1.88]) and anthracyclines antibiotics (OR 1.22, 95% CI [1.04, 1.42]) also had a significantly higher risk of having ≥ 1 dental health problem. Female survivors (OR 1.35, 95% CI [1.12, 1.62]), those older at follow-up (OR 1.05, 95% CI [1.04, 1.06]), those who ever smoked (OR 1.43, 95% CI [1.17, 1.74]), those who reported difficulty finding a dentist (OR 4.55, 95% CI [3.42, 6.05]), those who had dental visit in the past year (OR 1.39, 95% CI [1.11, 1.76]), and who were exposed to radiation therapy potentially exposing the oral cavity (OR 1.52, 95% CI [1.25, 1.84]) had higher risk of one or more soft tissue oral problem.

**Table 7:** Results of multivariable analysis of combined outcomes for survivors

Characteristic	At least one dental health issue			At least one soft tissue issue		
	OR	95% CI	*P* value	OR	95% CI	*P* value
Race						
Non-white	–	–				
White	1.25	1.02, 1.55	0.034			
Household income						
> $20, 000 per year	–	–				
< $20, 000 per year	1.19	0.96, 1.48	0.12			
Gender						
Male	–	–		–	–	
Female	1.29	1.12, 1.5	< 0.001	1.35	1.12, 1.62	0.002
Education						
Post high school	–	–		–	–	
No post high school	1.39	1.18, 1.63	< 0.001	0.89	0.73, 1.12	0.4
Dental insurance						
Yes	–	–		–	–	
No	0.94	0.78, 1.12	0.5	1.15	0.93, 1.42	0.2
Smoking status						
No	–	–		–	–	
Yes	1.79	1.53, 2.10	< 0.001	1.43	1.17, 1.74	< 0.001
Dental cleaning						
Yes	–	–				
No	1.1	0.92, 1.3	0.3			
Difficulty finding dentist						
No	–	–		–	–	
Yes	4.53	3.32, 6.25	< 0.001	4.55	3.42, 6.05	< 0.001
Patient ever received anthracyclines antibiotics						
No	–	–				
Yes	1.22	1.04,1.42	0.017			
Patient ever received classic alkylating agents						
No	–	–		–	–	
Yes	1.6	1.36, 1.88	< 0.001	0.97	0.80, 1.18	0.8
Patient ever had any potential impact on the oral body region due to radiation received						
No	–	–		–	–	
Yes	1.48	1.26, 1.72	< 0.001	1.52	1.25, 1.84	< 0.001
Age at last follow-up	1.02	1.01, 1.03	< 0.001	1.05	1.04, 1.06	< 0.001
Age at diagnosis	0.93	0.91, 0.94	< 0.001	1.01	0.99, 1.03	0.2
Dental visit						
No				–	–	
Yes				1.39	1.11, 1.76	0.005

OR, odds ratio; CI, confidence interval

## Discussion

The results of this study in a large, well characterized cohort, demonstrate that childhood cancer survivors have higher prevalence of oral dental abnormalities than do those without a childhood cancer history. Risk factors for adverse oral-dental outcomes include not only cancer treatment exposures, but also sociodemographic and dental access variables, factors amenable to interventions that help survivors navigate and gain access to necessary oral care following cancer therapy.

Cancer survivors had a 2- to 6-times greater prevalence of developmental tooth abnormalities, including unerupted teeth, misshapen teeth, enamel hypoplasia, and root malformation, as compared with matched controls. Receiving radiotherapy or chemotherapy at an early age (< 5 years old)—the period of prolific dental stem cell activity—increases the risk of odontogenic developmental abnormalities [4, 5, 13–15]. These abnormalities increase the need for palatal lift prosthesis, which we observed in the cancer survivors in our study. According to literature, enamel hypoplasia increases the risk of caries [16, 17]. However, the prevalence of > 5 caries did not differ between the survivor and control groups in our study. The survivors with enamel hypoplasia may have received proper dental care to prevent the number of caries, or alternatively the prevalence of enamel hypoplasia may have led to such extensive decay that resulted in tooth loss rather than caries, which is supported by the higher prevalence of missing > 6 teeth observed in survivors.

Xerostomia is associated with both radiotherapy and chemotherapy. Xerostomia is one of the most frequent complications after radiation therapy and is related to the cumulative dose of radiation affecting the salivary glands. Damage of the salivary glands decreases salivary flow, increases viscosity, and reduces saliva pH [18]. The current results demonstrate the significant association between radiotherapy and soft tissue abnormalities including xerostomia and severe gingivitis. Chemotherapy may also cause xerostomia in cancer survivors but the significant association between chemotherapy and xerostomia was not seen in the present results. Because more survivors experienced severe gingivitis and reported difficulty in finding a dentist, the lack of routine professional teeth cleanings or maintenance may also have contributed to their higher prevalence of severe gingivitis.

Higher income, higher educational attainment, female sex, and white race were all associated with more recent dental visits and teeth cleanings. These factors are contrary to experiencing problems finding a dentist and indicate potential disparities in access to oral health care. Because childhood cancer survivors experience more adverse dental outcomes than do those without cancer history, improving access to dental services by incorporating interventions at multiple levels throughout life is important for long-term survivors [19].

As an independent factor, we found that lower socioeconomic status (lower income and educational attainment) is associated with a higher prevalence of adverse dental outcomes. The role of socioeconomic status on oral health is well known [20]. Targeted programs for childhood cancer survivors with lower socioeconomic status to improve oral health are needed. Interestingly, we found that white and female survivors had more prevalent dental problems than did nonwhite and male survivors, in contrast with that reported in prior studies [21, 22]. Males generally have poorer oral health than do females because of poorer oral hygiene habits, ignored oral health, and less frequent dental visits [21]. However, these associations between gender and dental abnormalities of the current study are in alignment with the report of Childhood Cancer Survivor Study [5]. In the United States, ethnic minorities generally have poorer oral health, such as a high rate of caries and severe periodontal disease, when compared to whites [22]. The discrepancy between our findings and those of previous studies may be attributed to more consistent access to dental care among White and female childhood cancer survivors, resulting in greater awareness of their oral abnormalities and subsequent reporting of these abnormalities in the SJLIFE survey.

Our study is limited by the validity of self-reported oral abnormalities in cancer survivors and matched control subjects. For nonprofessionals, some abnormalities, such as missing teeth and the use of palatal lift prosthesis, are easier to recognize than are other abnormalities, such as enamel hypoplasia and root abnormalities. Therefore, caution must be exercised when comparing our reported prevalence of oral abnormalities to those in other studies that include a clinical validation. Cancer therapy can directly affect the development of oral tissues, resulting in oral diseases [23], and a history of cancer can negatively affect access to dental care, thereby increasing oral disease risk [1]. However, the casual association between cancer treatment and oral abnormalities must be confirmed in a prospective study with multiple time points. Additionally, we did not consider the prevalence of systemic diseases, such as diabetes and cardiovascular disease, that are associated with poor oral health in the general population. Future studies investigating associations between systemic health and oral health in cancer survivors are therefore needed.

## Conclusion

Childhood cancer survivors have a higher prevalence of oral-dental abnormalities than the controls without a cancer history. Cancer treatment, socioeconomic factors, and access to oral health care contribute to the prevalence of dental abnormalities. In summary, our analysis of comprehensive dental data obtained from childhood cancer survivors contributes to the research base of the various risk factors associated with adverse oral outcomes and the complications of cancer therapy late effects on oral abnormalities in long-term survivors. Management of the oral health for childhood cancer survivors is essential to prevent and ameliorate oral sequelae and improve overall quality of life.

## Data Availability

The raw data that support the findings of this study are available upon request at https://sjlife.stjude.org/data-sharing.html.
